# Supplementary material for: Metformin Is a Pyridoxal-5′-phosphate (PLP)-Competitive Inhibitor of SHMT2
Source: Cancers (Basel). 2021 Aug 9;13(16):4009. doi: 10.3390/cancers13164009 (PMC8393646; doi:10.3390/cancers13164009)
Supplement: Supplementary file 1 [file cancers-13-04009-s001.zip › cancers-1289388_supp_for_XML.docx]

Supplementary material

Metformin is a pyridoxal 5-phosphate (PLP)-competitive inhibitor of SHMT2


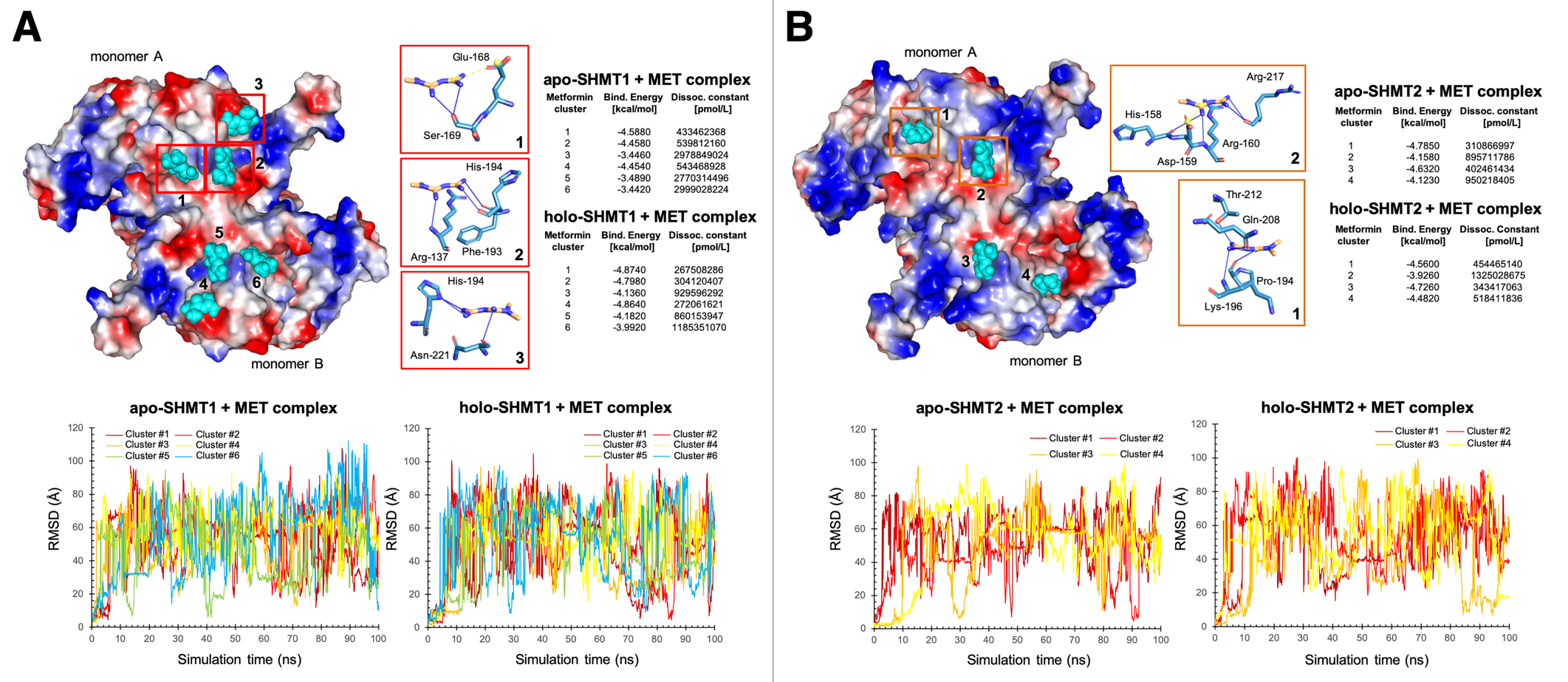


**Figure S1. Computational behavior of metformin at the SHMT dimer-to-dimer interface.** *Top panels.* Surface representation of the Scheme 1. (**A**) and SHMT2 (**B**) dimer-to-dimer interface showing the computationally predicted location of metformin (MET) clusters. Positions #1, #2, and #3 in SHMT1 monomer A are symmetrical to positions #6, #5, and #4 in SHMT1 monomer B. Positions #1 and #2 in SHMT2 monomer A are symmetrical to positions #4 and #3 in SHMT2 monomer B. Insets show the detailed interactions of the best poses of MET docked at each cluster using the PLIP algorithm, indicating the participating amino acids involved in the interaction and the type of interaction (hydrogen bonds, hydrophilic interactions, salt bridges, Π-stacking, etc). YASARA-based calculations of binding energy (ΔG, kcal/mol) and dissociation constants (pmol/L) for each MET cluster are also shown. Figures were prepared using PyMol 2.3 software. *Bottom panels.* The root-mean-square deviation (Å) of each MET heavy atom over the 0–100 ns time period of the molecular dynamics (MD) simulation, measured after superposing the protein on its reference structure, is shown for each cluster at the SHMT dimer-to-dimer interface in apo- and holo-SHMT/MET complexes.


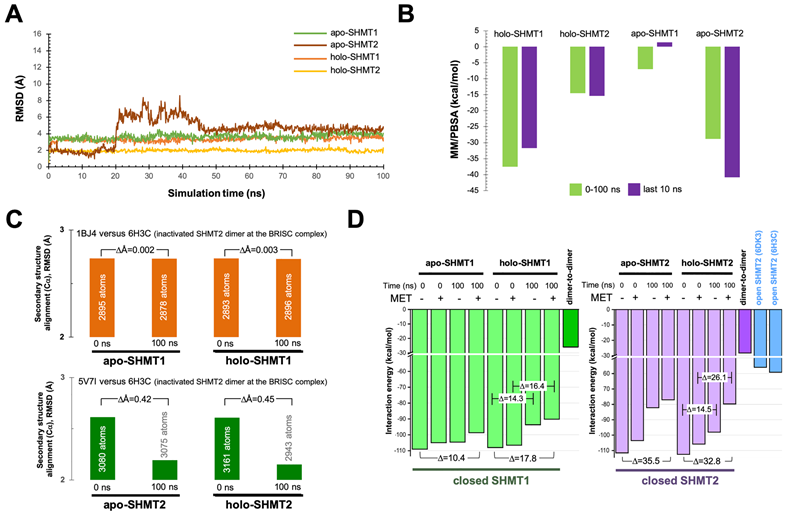


**Figure S2. Structure and stability of the SHMT-metformin complex. A.** The root-mean-square deviation (RMSD, Å) of each metformin (MET) heavy atom at the PLP-binding cavity over the 0–100 ns time period of a molecular dynamics (MD) simulation, measured after superposing the protein on its reference structure, is shown for each apo- and holo-SHMT/MET complex. **B.** Molecular Mechanics Poisson–Boltzmann Surface Area (MM/PBSA) free energy analysis of apo- and holo-SHMT1/2 forming complexes with MET using YASARA v19.9.17 software. In each case, the best docked complex as the initial conformation for MD simulation followed by 1000 snapshots (100 ns) obtained from the MD trajectory was employed to calculate the values of free energy binding of MET. Additionally, the average value calculated for the last 100 snapshots (10 ns) is also displayed. YASARA-calculated binding energy provides positive values when the predicted binding is strong and stable whereas negative values indicate no binding. Figures were prepared using PyMol 2.3 software. **C.** RMSD values calculated after aligning the alpha carbons of either the closed apo- and holo-SHMT1/metformin complex (1BJ4, *top*) or the closed apo- and holo-SHMT2/metformin complex (5V7I, *bottom*) before and after 100 ns of MD simulations with those of the inactive SHMT2 dimer present in the BRISC-SHMT (6H3C, [36]). **D.** FoldX-calculated interaction energy (kcal/mol) (Delgado et al., 2019) between each monomer of the closed apo- and holo-SHMTs in the absence or presence of docked MET at the PLP binding cavity before (0 ns) and after (100 ns) of MD simulations. This panel includes also the interaction energies calculated between both dimers of a closed SHMT2 tetramer and between each monomer of the two open SHMT2 conformations 6DK3 and 6H3C [36].
