# Supplementary figures and images for: Metformin Is a Pyridoxal-5′-phosphate (PLP)-Competitive Inhibitor of SHMT2
Source: Cancers (Basel). 2021 Aug 9;13(16):4009. doi: 10.3390/cancers13164009 (PMC8393646; doi:10.3390/cancers13164009)

Uncropped images (immuboblotting Figure 6)

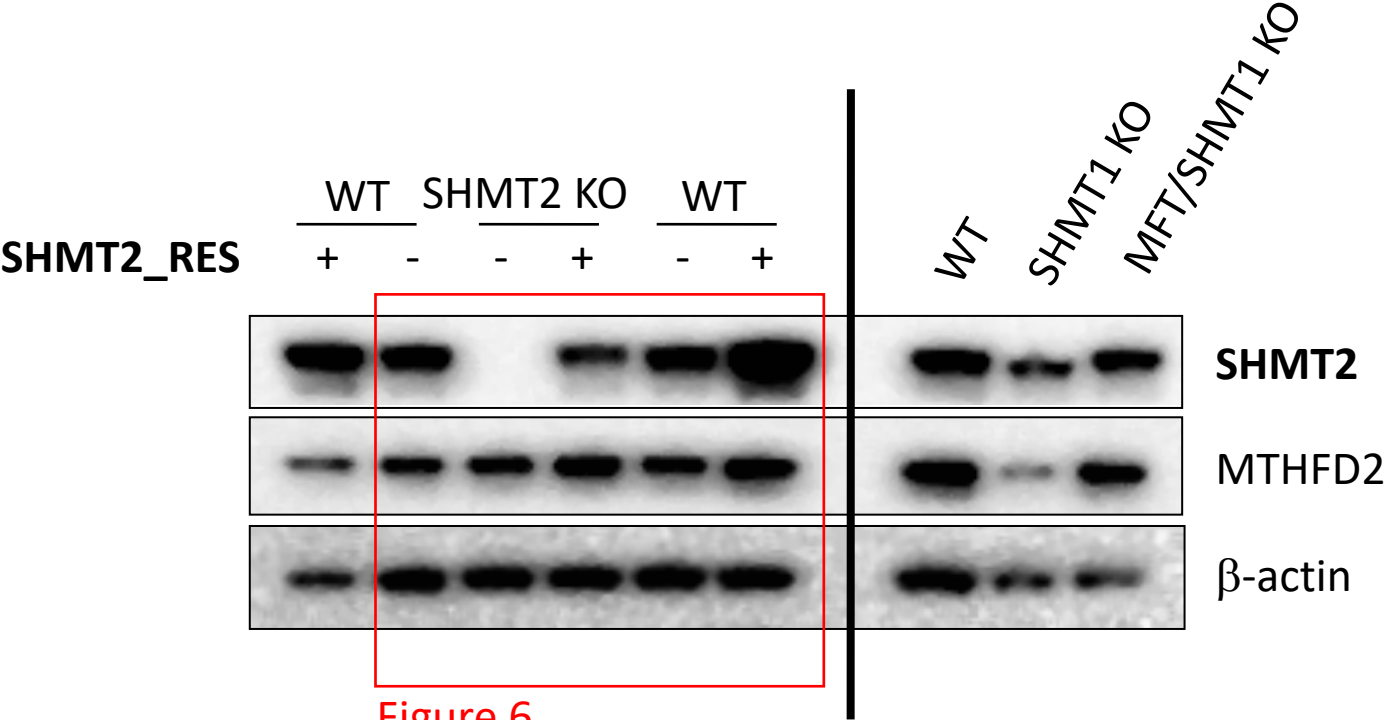

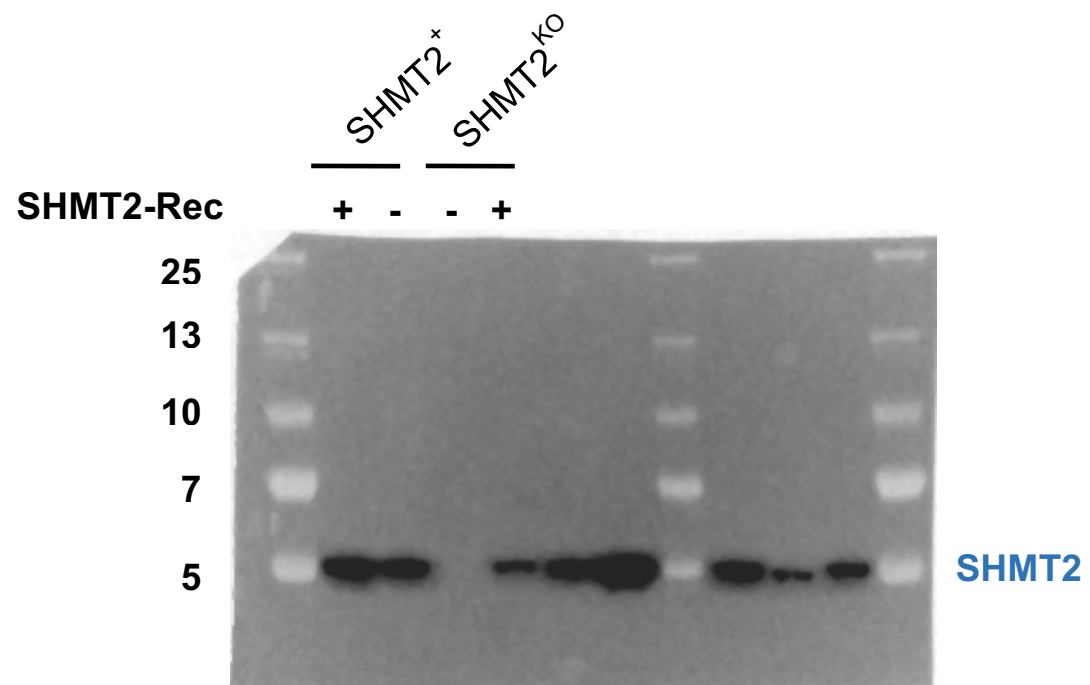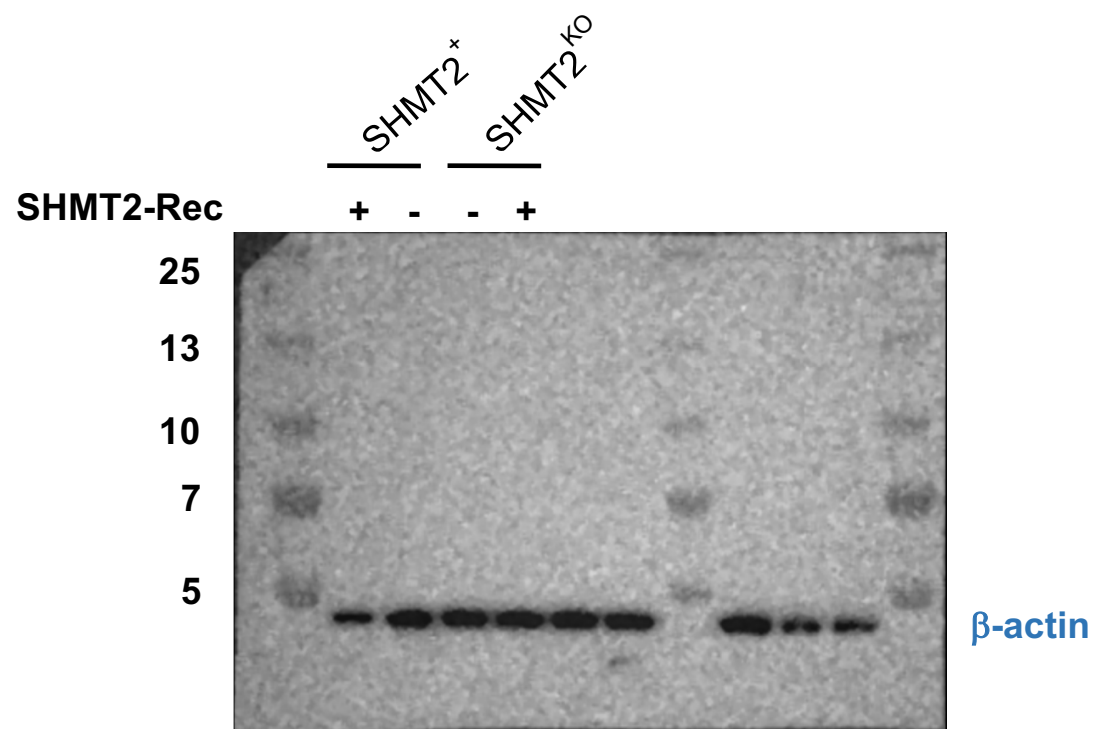

Supplement: Supplementary file 1 [file cancers-13-04009-s001.zip › cancers-1289388_uncroppedWB.pdf]
